# Supplementary material for: Polygenic and developmental profiles of autism differ by age at diagnosis
Source: Nature. 2025 Oct 1;646(8087):1146–55. doi: 10.1038/s41586-025-09542-6 (PMC12571882; doi:10.1038/s41586-025-09542-6)
Supplement: Supplementary file 2 — Reporting Summary [file 41586_2025_9542_MOESM2_ESM.pdf]

Corresponding author(s): Warrier
Last updated by author(s): Jul 17, 2025

Reporting Summary

Nature Portfolio wishes to improve the reproducibility of the work that we publish. This form provides structure for consistency and transparency in reporting. For further information on Nature Portfolio policies, see our Editorial Policies and the Editorial Policy Checklist.

Statistics

For all statistical analyses, confirm that the following items are present in the figure legend, table legend, main text, or Methods section.

n/a	Confirmed	
☐	☒	The exact sample size (*n*) for each experimental group/condition, given as a discrete number and unit of measurement
☐	☒	A statement on whether measurements were taken from distinct samples or whether the same sample was measured repeatedly
☐	☒	The statistical test(s) used AND whether they are one- or two-sided<br>*Only common tests should be described solely by name; describe more complex techniques in the Methods section.*
☐	☒	A description of all covariates tested
☐	☒	A description of any assumptions or corrections, such as tests of normality and adjustment for multiple comparisons
☐	☒	A full description of the statistical parameters including central tendency (e.g. means) or other basic estimates (e.g. regression coefficient) AND variation (e.g. standard deviation) or associated estimates of uncertainty (e.g. confidence intervals)
☐	☒	For null hypothesis testing, the test statistic (e.g. *F*, *t*, *r*) with confidence intervals, effect sizes, degrees of freedom and *P* value noted<br>*Give P values as exact values whenever suitable.*
☒	☐	For Bayesian analysis, information on the choice of priors and Markov chain Monte Carlo settings
☒	☐	For hierarchical and complex designs, identification of the appropriate level for tests and full reporting of outcomes
☐	☒	Estimates of effect sizes (e.g. Cohen's *d*, Pearson's *r*), indicating how they were calculated

Our web collection on statistics for biologists contains articles on many of the points above.

Software and code

Policy information about availability of computer code

Data collection	No separate software was used to collect data.
Data analysis	Lavaan (LGCM) (v.0.6-19) : https://lavaan.ugent.be/tutorial/growth.html<br>lcmm(GMM), v 2.2.1: https://github.com/CecileProust-Lima/lcmm<br>Softimpute (v.1.4-1): https://cran.r-project.org/web/packages/softImpute/softImpute.pdf<br>Misty (v 0.6.8) : https://cran.r-project.org/web/packages/misty/index.html<br>PRScs (v.1.1.0): https://github.com/getian107/PRScs<br>fastGWA and GCTA (v.1.94.1): https://yanglab.westlake.edu.cn/software/gcta/#Overview<br>GenomicSEM (v.0.0.5): https://github.com/GenomicSEM/GenomicSEM<br>LDSC (v1.0.1): https://github.com/bulik/ldsc<br>KING (v.2.3.2): https://www.kingrelatedness.com/manual.shtml<br>Plink 2.0: https://www.cog-genomics.org/plink/2.0/<br>GENESIS (v2.22.2): https://github.com/UW-GAC/GENESIS<br>Lme4 (v.1.1.27.1): https://github.com/lme4/lme4/<br>Logistf (v.1.24): https://cran.r-project.org/web/packages/logistf/index.html<br>Ebal (v 0.1-8): https://cran.r-project.org/web/packages/ebal/ebal.pdf<br>Relaimpo (v2.2-7): https://cran.r-project.org/web/packages/relaimpo/relaimpo.pdf<br><br>SPARK quality control, imputation and GWAS: https://github.com/vwarrier/SPARK_iWES2_imputation/<br>Bespoke genetic analyses code: https://github.com/vwarrier/autism_agediagnosis/

For manuscripts utilizing custom algorithms or software that are central to the research but not yet described in published literature, software must be made available to editors and reviewers. We strongly encourage code deposition in a community repository (e.g. GitHub). See the Nature Portfolio guidelines for submitting code & software for further information.

Data

Policy information about availability of data

All manuscripts must include a data availability statement. This statement should provide the following information, where applicable:

- Accession codes, unique identifiers, or web links for publicly available datasets
- A description of any restrictions on data availability
- For clinical datasets or third party data, please ensure that the statement adheres to our policy

SPARK autism GWAS: https://bitbucket.org/steinlabunc/spark_asd_sumstats/src
Finngen autism GWAS: https://www.finngen.fi/en/access_results
iPSYCH autism GWAS (unstratified, sex-stratified and age at diagnosis stratified, age at diagnosis) can be obtained from Anders Borglum and Jakob Grove.
Psychiatric GWAS summary stats: https://pgc.unc.edu/for-researchers/download-results/
GWAS educational attainment: https://thessgac.com/papers/
GWAS cognitive aptitude: https://cncr.nl/research/summary_statistics/
For ALSPAC, the study website contains details of all the data that is available through a fully searchable data dictionary and variable search tool": http://www.bristol.ac.uk/alspac/researchers/our-data/
For MCS, data can be obtain after application through the UK Data Service: https://beta.ukdataservice.ac.uk/datacatalogue/series/series?id=2000031
Summary statistics for the SPARK-based age at diagnosis GWAS, and the age at diagnosis stratified GWAS generated from the genomicSEM models are available here: https://figshare.com/articles/dataset/Summary_statistics_for_Polygenic_and_developmental_profiles_of_autism_differ_by_age_at_diagnosis/29566052.

Research involving human participants, their data, or biological material

Policy information about studies with human participants or human data. See also policy information about sex, gender (identity/presentation), and sexual orientation and race, ethnicity and racism.

Reporting on sex and gender	We use the term sex throughout, which primarily refers to sex assigned at birth.
Reporting on race, ethnicity, or other socially relevant groupings	We restrict our genetic analyses to individuals of genetically inferred European ancestries. For trajectory modelling in birth cohorts, we do not exclude individuals based on informant-reported ancestry, and include informant-reported ancestry as a covariate in some models.
Population characteristics	Data from existing human population cohorts/databases were used, including from birth cohorts. The four birth cohorts are: Millennium Cohort Study (year of birth - 2000/2001), Growing up in Ireland (year of birth - 1998), Longitudinal Study of Australian Children - Kindergarten cohort (Year of birth - 1999 - 2000) and Birth cohort (Year of birth - 2003 - 2004). In these birth cohort, a subset of the children were autistic. MCS - 238, GUI - 109, LSAC-K - 89, LSAC-B - 129). For PGS analyses, we also included data from ALSPAC (Year of birth 1990 - 1991).<br><br>We included data from two cohorts of autistic individuals and their families. These were the US-based SPARK cohort, including a Discovery subset (N = 18,809, median age at autism diagnosis -= 4.0 years), and a Replication subset (N = 9,701, median age at autism diagnosis = 5.0 years), and the Danish based iPSYCH cohort (N = 18,965, median age at autism diagnosis = 4.0).<br><br>Detailed population characteristics of the birth cohorts, and the cohorts used for the GWAS analyses are provided in the relevant section of the Methods or Supplementary Information.
Recruitment	Data from existing human population cohorts/databases were used, including from birth cohorts. Details of recruitment and population characteristics are provided in the relevant section of the Methods or Supplementary Information.
Ethics oversight	Data for existing cohorts were collected based on ethical approval from local IRBs. Analyses of de-identified data from cohorts was approved by the Cambridge Human Biology Research Ethics Committee.

Note that full information on the approval of the study protocol must also be provided in the manuscript.

Field-specific reporting

Please select the one below that is the best fit for your research. If you are not sure, read the appropriate sections before making your selection.

☒ Life sciences ☐ Behavioural & social sciences ☐ Ecological, evolutionary & environmental sciences

For a reference copy of the document with all sections, see nature.com/documents/nr-reporting-summary-flat.pdf

Life sciences study design

All studies must disclose on these points even when the disclosure is negative.

Sample size	As the study relied on existing data, we used the maximum available sample after quality control. No a priori sample size calculation was conducted.
Data exclusions	Data were excluded after phenotypic or genetic quality control, and this varied by cohorts. Further details are provided in Methods.
Replication	Where possible, all analyses were conducted in at least two cohorts to assess the replicability and generalisability of the findings. Trajectory modelling was conducted in four birth cohorts. Genetic analyses of autistic individuals were conducted in two cohorts - SPARK and iPSYCH. Genetic analyses of the general population were conducted in two cohorts - MCS and ALSPAC, with additional support from MoBa. In addition, we included a replication cohort from SPARK, which we analysed only after the first version of the manuscript was submitted and reviewed. This was available only after the initial submission of the manuscript. Details are provided in the manuscript. All effects were in the consistent direction between the original Discovery and the Replication cohorts, and meta-analysis of the two datasets increased the statistical significance of the findings, indicating that replication was largely successful.
Randomization	No randomisation was conducted as this is an observational study.
Blinding	No blinding was conducted as this is an observational study.

Reporting for specific materials, systems and methods

We require information from authors about some types of materials, experimental systems and methods used in many studies. Here, indicate whether each material, system or method listed is relevant to your study. If you are not sure if a list item applies to your research, read the appropriate section before selecting a response.

Materials & experimental systems

n/a	Involved in the study	
☒	☐	Antibodies
☒	☐	Eukaryotic cell lines
☒	☐	Palaeontology and archaeology
☒	☐	Animals and other organisms
☒	☐	Clinical data
☒	☐	Dual use research of concern
☒	☐	Plants

Methods

n/a	Involved in the study	
☒	☐	ChIP-seq
☒	☐	Flow cytometry
☒	☐	MRI-based neuroimaging

Plants

Seed stocks	N/A
Novel plant genotypes	N/A
Authentication	N/A
